# Dorsal subthalamic nucleus targeting in deep brain stimulation: microelectrode recording versus 7-Tesla connectivity

**DOI:** 10.1093/braincomms/fcad298

**Published:** 2023-11-11

**Authors:** Naomi I Kremer, Mark J Roberts, Wouter V Potters, José Dilai, Varvara Mathiopoulou, Niels Rijks, Gea Drost, Teus van Laar, J Marc C van Dijk, Martijn Beudel, Rob M A de Bie, Pepijn van den Munckhof, Marcus L F Janssen, P Richard Schuurman, Maarten Bot

**Affiliations:** Department of Neurosurgery, Amsterdam University Medical Centers, Amsterdam 1105 AZ, The Netherlands; Department of Neurosurgery, University of Groningen, University Medical Center Groningen, Groningen 9713 GZ, The Netherlands; Faculty of Psychology and Neuroscience, Maastricht University, Maastricht 6211 LK, The Netherlands; Department of Neurology and Clinical Neurophysiology, Amsterdam University Medical Centers, Amsterdam 1105 AZ, The Netherlands; Department of Neurology and Clinical Neurophysiology, Amsterdam University Medical Centers, Amsterdam 1105 AZ, The Netherlands; Department of Neurosurgery, Amsterdam University Medical Centers, Amsterdam 1105 AZ, The Netherlands; Department of Neurosurgery, Amsterdam University Medical Centers, Amsterdam 1105 AZ, The Netherlands; Department of Neurosurgery, University of Groningen, University Medical Center Groningen, Groningen 9713 GZ, The Netherlands; Department of Neurology, University of Groningen, University Medical Center Groningen, Groningen 9713 GZ, The Netherlands; Department of Neurology, University of Groningen, University Medical Center Groningen, Groningen 9713 GZ, The Netherlands; Department of Neurosurgery, University of Groningen, University Medical Center Groningen, Groningen 9713 GZ, The Netherlands; Department of Neurology and Clinical Neurophysiology, Amsterdam University Medical Centers, Amsterdam 1105 AZ, The Netherlands; Department of Neurology and Clinical Neurophysiology, Amsterdam University Medical Centers, Amsterdam 1105 AZ, The Netherlands; Department of Neurosurgery, Amsterdam University Medical Centers, Amsterdam 1105 AZ, The Netherlands; Department of Clinical Neurophysiology, Maastricht University Medical Center, Maastricht 6229 HX, The Netherlands; Department of Neurosurgery, Amsterdam University Medical Centers, Amsterdam 1105 AZ, The Netherlands; Department of Neurosurgery, Amsterdam University Medical Centers, Amsterdam 1105 AZ, The Netherlands

**Keywords:** deep brain stimulation, Parkinson’s disease, subthalamic nucleus, connectivity, targeting

## Abstract

Connectivity-derived 7-Tesla MRI segmentation and intraoperative microelectrode recording can both assist subthalamic nucleus targeting for deep brain stimulation in Parkinson’s disease. It remains unclear whether deep brain stimulation electrodes placed in the 7-Tesla MRI segmented subdivision with predominant projections to cortical motor areas (hyperdirect pathway) achieve superior motor improvement and whether microelectrode recording can accurately distinguish the motor subdivision. In 25 patients with Parkinson’s disease, deep brain stimulation electrodes were evaluated for being inside or outside the predominantly motor-connected subthalamic nucleus (motor-connected subthalamic nucleus or non-motor-connected subthalamic nucleus, respectively) based on 7-Tesla MRI connectivity segmentation. Hemi-body motor improvement (Movement Disorder Society Unified Parkinson’s Disease Rating Scale, Part III) and microelectrode recording characteristics of multi- and single-unit activities were compared between groups. Deep brain stimulation electrodes placed in the motor-connected subthalamic nucleus resulted in higher hemi-body motor improvement, compared with electrodes placed in the non-motor-connected subthalamic nucleus (80% versus 52%, *P* < 0.0001). Multi-unit activity was found slightly higher in the motor-connected subthalamic nucleus versus the non-motor-connected subthalamic nucleus (*P* < 0.001, receiver operating characteristic 0.63); single-unit activity did not differ between groups. Deep brain stimulation in the connectivity-derived 7-Tesla MRI subthalamic nucleus motor segment produced a superior clinical outcome; however, microelectrode recording did not accurately distinguish this subdivision within the subthalamic nucleus.

## Teaser

Kremer *et al*. report that deep brain stimulation in the connectivity-derived 7-Tesla MRI subthalamic nucleus motor segment produces superior clinical outcomes, compared with stimulation in the non-motor-connected subthalamic nucleus. Multi- and single-unit activities, derived from microelectrode recordings, were not able to reliably distinguish the motor segment within the subthalamic nucleus.

## Graphical Abstract

**Graphical Abstract:**
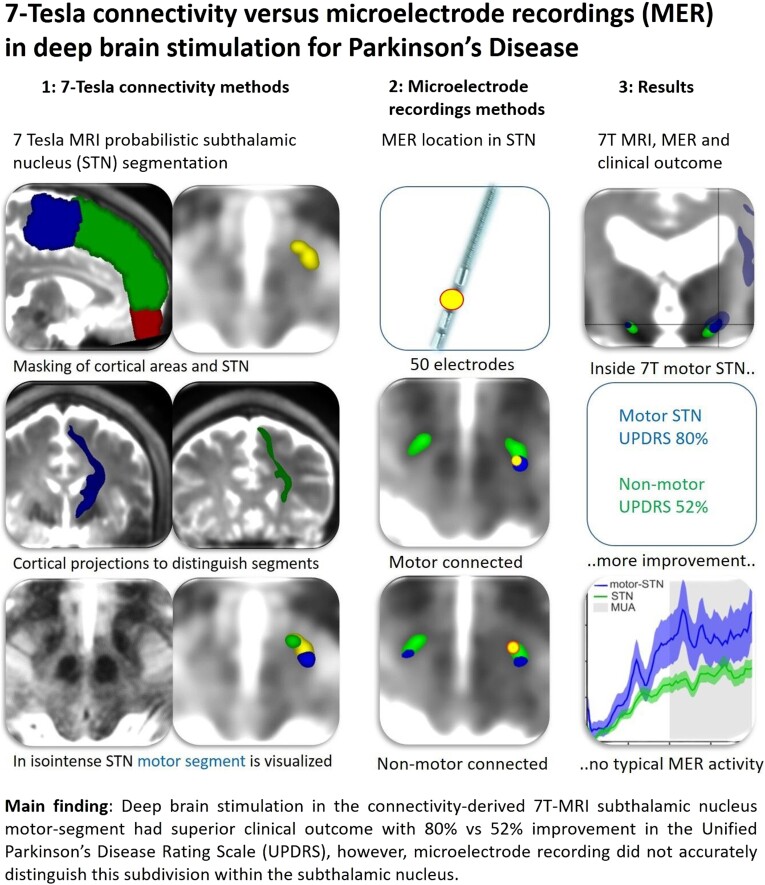

## Introduction

Deep brain stimulation of the subthalamic nucleus (STN-DBS) is an effective treatment for patients with advanced Parkinson’s disease. The dorsal STN contains the highest density of projections to cortical motor areas (hyperdirect pathway) and is considered the optimal target for DBS.^1-7^ Size and orientation challenge dorsal STN visualization with T_2_-weighted MRI; therefore, microelectrode recording (MER) is regularly used for intraoperative electrophysiological confirmation.^8,9^ MER is able to identify the dorsal and ventral STN borders by differences in the high-frequency power of multi-unit activity (MUA).^10,11^ Further mapping of the dorsal STN motor area can be achieved by measuring beta-oscillatory activity, derived from either local field potentials or MUA of sufficient recording length.^12,13^ Recently, diffusion-weighted MRI techniques have been applied to identify separate functional STN subdivisions, enabling non-invasive visualization of the motor-STN for DBS surgery.^1^ In this study, we evaluate whether DBS electrodes placed in the 7-Tesla (7 T) MRI connectivity–derived STN subdivision with predominant (direct) projections to cortical motor areas achieve a superior clinical outcome and whether MER can accurately distinguish the motor subdivision.^7^

## Materials and methods

All patients with Parkinson’s disease who underwent STN-DBS at the Amsterdam University Medical Centers between January 2019 and February 2020 and for whom preoperative 7 T MRI (T_2_ and diffusion-weighted sequences) and MER were available were included in the study. Details of the surgical procedure and connectivity-derived STN segmentation were provided previously.^7,14^ Patients underwent stereotactic surgery either under general or local anaesthesia, all guided by MER. In patients under general anaesthesia, we used remifentanil (25–33 µg/kg/h per continuous infusion) and propofol (6–10 mg/kg/h per continuous infusion), together with an ultrashort-acting muscle relaxant for endotracheal intubation. Propofol was stopped 20 min before starting MER, to allow STN activity to return. MER was started 5–6 mm above the intended target and advanced with 0.5 mm steps to 2–3 mm below the target. One to three simultaneous tracks were performed, with the planned trajectory as the central channel and additional lateral and anterior channels at a 2 mm distance from the central channel. Propofol cessation lasted maximally 45 min, while high-dose remifentanil (50  µg/kg/h per continuous infusion) was continued to ensure that wake-up did not occur. Boston DBS electrodes were bilaterally implanted in the track with the longest section of, or the most powerful, subthalamic activity signal (typical broadening of background activity with a tonic and irregular discharge pattern and an occasional burst). Immediately after electrode implantation on the second side, propofol was resumed. In patients under local anaesthesia, the electrode implantation was preceded by additional macroelectrode stimulation to test for symptom reduction and stimulation-induced side effects. These patients were in off-medication state (at least 12 h withdrawal of parkinsonian medication) during the surgery. Intraoperative cone-beam CT was used to assess the electrode position. Implantation of the extension wires and the internal pulse generator was performed under general anaesthesia in the same session.

Connectivity-derived STN segmentation was performed in the postoperative phase. The segmentations were conducted using our previously published protocol (ensuring standardized acquisition, masking and connectivity visualization).^7^ We manually delineated (masked) the STN in native space, co-registering and superimposing the 7 T T_2_-weighted MRI to the diffusion-weighted image in ITK-SNAP v3.8. Voxels that contained at least 50% STN (hypo-)intensity were included. In FSL [FMRIB (Functional Magnetic Resonance Imaging of the Brain) Software Library, Oxford, UK], we identified and masked three major STN projections: one from STN to primary and supplementary motor cortex (motor), a second to the prefrontal cortex (associative) and a third to the basofrontal cortex (limbic). Running PROBTRACKX in FSL (default parameters), using ipsilateral cortical masks as classification targets, resulted in an STN segmentation derived from direct connectivity between STN and the three specific cortical areas. We considered voxels to be connected to that specific cortical area if at least 20% of its probabilistic tracks connected the two structures (i.e. STN and cortical area). This threshold was preferred over a winner-takes-all approach, because one voxel might contain several white matter tracks.^15^ Motor-connected STN segments were assigned to the colour blue and non-motor-connected STN was assigned to the colour green. The active DBS contact location was used to create two groups: situated either inside the predominantly motor-connected STN subdivision (mc-STN, blue) or in the non-motor-connected STN (nmc-STN, green). The active DBS contact location (monopolar stimulation was applied to all patients) on postoperative CT was plotted on the corresponding segmented STN to determine its location relative to the predominantly motor-connected subdivision. None of the active electrode contacts were located in both mc-STN and nmc-STN.

Hemi-body motor improvement was assessed at baseline and after a 6-month follow-up using the Movement Disorder Society Unified Parkinson’s Disease Rating Scale, Part III (MDS-UPDRS III), both in off-medication state (after withdrawal of parkinsonian medication for at least 12 h). Hemi-body scores were calculated by summating Items 3–8 and 15–17. The postoperative motor evaluation was part of the usual clinical follow-up, and raters were blinded to the exact location of the electrodes. DBS stimulation parameters used for assessing the stimulation effect are described in Supplementary Table 1. The Research Ethical Board reviewed the study protocol and waived formal protocol approval under the Dutch Medical Research Involving Human Subjects Act.

The MER track corresponding to the implanted DBS electrode was used for analysis. Recordings had a 20 kHz sampling rate, were low-pass-filtered at 5 kHz by the clinical software (ISIS MER software v3.2.1.0; Inomed GmbH) and lasted ∼8 s. Custom-build MATLAB scripts and the FieldTrip toolbox^16^ were used for MUA and single-unit activity (SUA) offline data analysis. All signals were manually cropped to exclude movement artefacts and filtered with a sixth-order 160 Hz high-pass and 5000 Hz low-pass filter followed by a notch filter (50 Hz and harmonics).

We identified MUA, representing a combined activity of neurons near the microelectrode tip, as the mean of the power-spectral density of the signal within the bandwidth of 300–500 Hz. Prior to this calculation, periods of high noise were automatically identified by calculating the root mean square of the filtered data cut into 50 ms sections. Sections in which this value exceeded the mean by three standard deviations were discarded. The remaining data were then cut into one quarter overlapping sections of 250 ms, from which the power-spectral density was calculated with a multi-taper method. To account for differences between recording tracks (tissue and electrode impedance), we defined MUA from power in the STN, normalized by that of a baseline (non-STN) site.

To identify SUA, representing a single firing neuron, we identified periods of stable recording and set a threshold for spike identification. Spike shapes were selected using principal component analysis and k-means clustering. Single units were selected and confirmed by an inspection of the autocorrelation (<5% of spikes with a 3 ms gap between spikes). We calculated firing rate (spikes/s) and coefficient of variation, calculated as the standard deviation of the inter-spike interval divided by the mean. Bursts were automatically detected using the algorithm advanced by Bakkum *et al*.^17^ with *N* spikes per burst set to 8. This setting yielded burst parameters quantitatively similar to those of previous literature, i.e. at a burst length of ∼100 ms, with spikes occurring within bursts (burst index) of ∼40%.^18^ We calculated the burst rate, mean burst length, mean spikes per burst and burst index.

### Statistical analysis

Recording sites in the mc-STN were compared with those in the nmc-STN. For MUA, we performed two analyses: the main analysis and the sliding window analysis. An ANOVA with factors AREA (mc-STN/nmc-STN) and (anonymous) patient-ID (25 levels) as a random factor^19,20^ was performed for both analyses. The shared within-subject variance was accounted for by the random factor (anonymous patient-ID) in the ANOVA. For the main analysis, we used the three recordings per hemisphere covering the active DBS electrode contact site [MER is performed in 0.5 mm steps, the active contact length is 1.5 mm (Vercise Cartesia directional lead; Boston Scientific)], yielding six sites per patient (three sites per hemisphere), which could represent either six mc-STN sites, six nmc-STN sites or three sites of each area, depending on the implantation of the patient. For the sliding window analysis, to test the spatial specificity of the difference between MUA in mc-STN and nmc-STN, we repeated the main analysis, however now sliding along the entire MER trajectory (consisting of 16–25 recordings in total, with each trajectory aligned to the location of the active site), rather than only the three sites corresponding to the active contact site. The two-factor ANOVA (AREA and patient-ID as a random factor) was performed at each relative depth site (two sites were included per patient, one for each hemisphere) and corrected for multiple comparisons using a cluster-based permutation test. This analysis gives high spatial specificity, but it has lower statistical power than the main analysis. Therefore, for comparison with our main analysis, we also used a sliding window of three sites per hemisphere per relative depth, that is, at each depth, we included data from that depth and from the immediate neighbouring depths. We used receiver operating characteristic (ROC) analysis to quantify the reliability MUA as a metric for discriminating mc-STN from nmc-STN, including for each hemisphere the three recording sites corresponding to the active DBS electrode contact. For SUA, analyses were performed on the three recordings covering the active contact site. Differences in SUA and recorded STN length were tested with two-sample *t*-tests. Differences in hemi-body motor improvement were tested with Welch’s *t*-test for unequal variance. Statistical analyses were performed in MATLAB and IBM SPSS Statistics version 28. A two-sided alpha of 0.05 was used as the level of significance.

## Results

**Figure 1:**
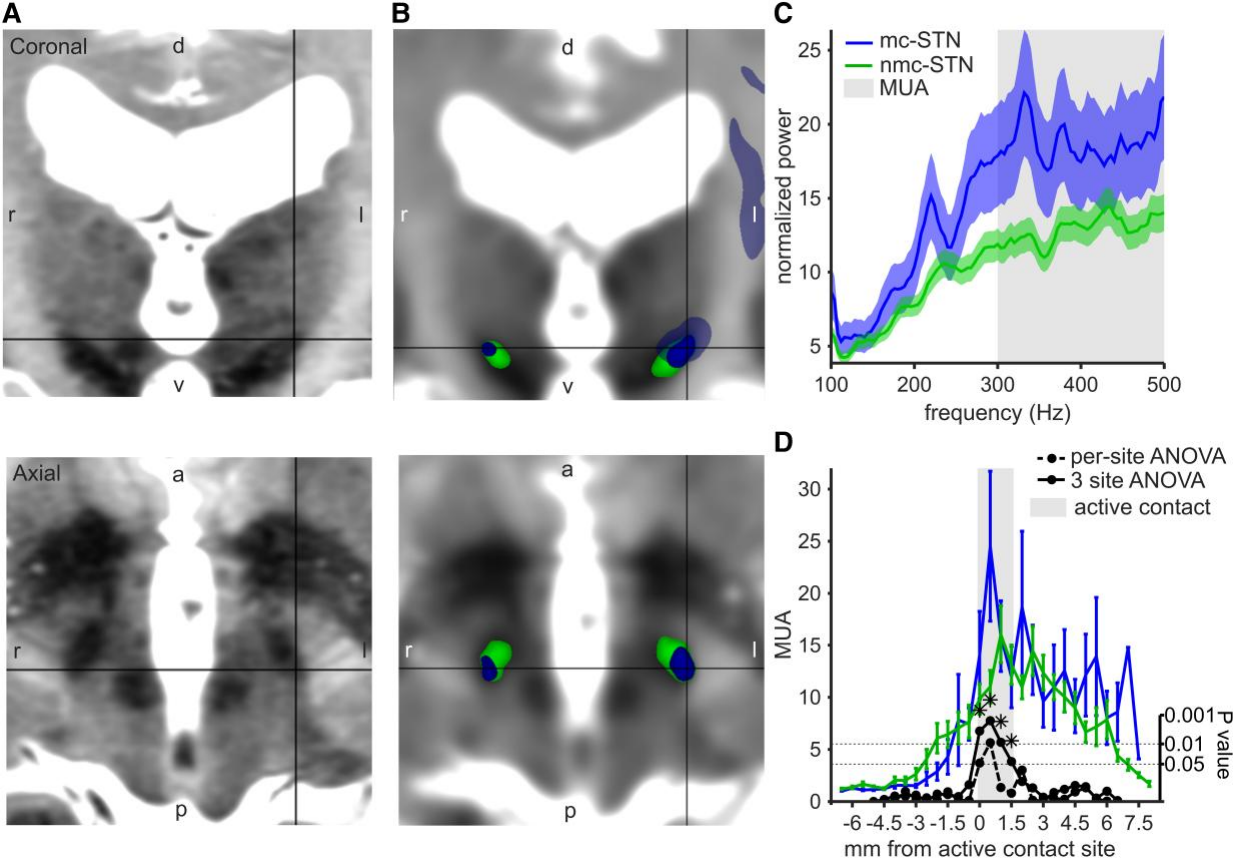
**Connectivity-derived STN segmentation and MER analysis.** (**A**) 7 T T_2_ MRI of an example patient showing the STN in coronal and axial planes. d: dorsal, v: ventral, l: left, r: right. (**B**) 7 T T_2_ MRI registered to the diffusion MRI and the connectivity-derived STN segmentation; motor-connected STN (mc-STN, blue) with projections to the cortical motor areas (transparent blue) and non-motor-connected STN (nmc-STN, green) in the same example patient. The black pointer indicates the dorsal STN motor subdivision. Images are aligned to the commissural line. (**C**) Baseline-corrected power spectrum for mc-STN and nmc-STN. MUA calculated as mean power >300 Hz. The solid line showing mean and the shading showing standard error. (**D**) Mean MUA at each recording site, aligned to the active location site (grey shading). Mc-STN in blue and nmc-STN in green. The error bars show standard error. Stippled black line: 1-log *P*-value from ANOVA calculated at each site. The solid black line, which is the same for ANOVA, calculated in a three-site sliding window. The stars denote significant differences after multiple comparison correction. mc, motor-connected; nmc, non-motor-connected; STN, subthalamic nucleus; MER, microelectrode recording; MUA, multi-unit activity. Sample size MUA: total recordings *n* = 149 (25 patients, 3 recordings per hemisphere covering the active DBS electrode contact site, for 1 patient the most dorsal active contact site was used, which resulted in 2 recordings for that hemisphere); motor-connected STN recordings *n* = 27; non-motor-connected STN recordings *n* = 122.

Twenty-five patients (8 women, 17 men; mean age, 60.6 ± 10.1 years) were included in the study. All patients underwent bilateral STN-DBS, with 19 procedures performed under general anaesthesia. Nine electrodes (18%) had active contacts in the mc-STN; 41 electrodes (82%) in the nmc-STN (Fig. 1A and B). Active contacts located in the mc-STN resulted in a higher hemi-body motor improvement than contacts located in the nmc-STN [80% versus 52% decrease in off-medication MDS-UPDRS III, *t*(27.7) = 4.89, *P* < 0.0001]. There were no differences in the therapeutic parameters used for electrodes in the mc-STN and the nmc-STN [Supplementary Table 1; amplitude *t*(48) = 0.20, *P* = 0.85, frequency *t*(48) = −0.09, *P* = 0.93]. Electrodes located in the nmc-STN were not associated with more side effects at higher intensities than those located in the mc-STN [Supplementary Table 1; *t*(28) = −1.12, *P* = 0.27].

Considering the three sites corresponding to the active contact at each trajectory, MUA was higher in the mc-STN compared with the nmc-STN (Fig. 1C, Table 1). The corresponding area under the curve (AUC) of the ROC was 0.63. When considering MUA along the entire trajectory (Fig. 1D), we found significant differences only at the sites corresponding to the active contact (three-site sliding window ANOVA, cluster-based permutation test, *P* = 0.02). No differences between mc-STN and mnc-STN sites based on the 28 well-isolated single units were found (Table 1). Recorded STN length did not differ between trajectories that included mc-STN and those that included nmc-STN [*t*(48) = 0.20, *P* = 0.84]. MUA at the active contact site did not differ between patients with local and general anaesthesia [two-sample *t*-test, *t*(147) = 1.30, *P* = 0.19] but was found to decrease with patients’ age (*R*^2^ = 0.0284, *F* = 4.3045, *P* = 0.04).

**Table 1:** MER characteristics

Multi-unit activity	Combined (*n* = 149)	Motor-connected STN (*n* = 27)	Non-motor-connected STN (*n* = 122)
Normalized MUA*	14.5 ± 15.9	18.3 ± 15.9	13.6 ± 15.8

Single-unit activity	Combined (*n* = 28)	Motor-connected STN (*n* = 5)	Non-motor-connected STN (*n* = 23)
Firing rate (spikes/s)	32.1 ± 18.6	30.7 ± 13.1	32.4 ± 19.8
Coefficient of variation	1.83 ± 0.90	1.48 ± 0.63	1.91 ± 0.94
Burst rate (bursts/s)	0.97 ± 0.79	1.01 ± 0.97	0.96 ± 0.78
Burst length (s)	0.15 ± 0.07	0.13 ± 0.03	0.16 ± 0.07
Spikes/burst	14.6 ± 7.92	11.3 ± 2.36	15.2 ± 8.50
Burst index (%)	40 ± 23	0.31 ± 0.16	0.42 ± 0.24

Data are presented as mean ± standard deviation. ANOVA was used for MUA analysis. Motor-connected STN versus non-motor-connected STN: *F*(1,123) = 10.38, *P* = 0.002. Patient-ID random factor: F(24,123) = 2.61, P = 0.0003. Two-sample *t*-tests were used for SUA analysis; no comparison showed a significant difference. STN, subthalamic nucleus; MUA, multi-unit activity; *n*, sample size. *P < 0.05.

## Discussion

In this study, 7 T MRI connectivity was used to identify the STN subdivision with predominant projections to cortical motor areas. Active DBS electrode contacts located in this subdivision showed significantly better hemi-body motor improvement. However, MER based on MUA and SUA was not able to reliably distinguish the connectivity-derived mc-STN.

The current study is in line with the finding that MER can readily distinguish STN from zona incerta (dorsal border) or substantia nigra (ventral border) using MUA,^10,11^ even under general anaesthesia,^14^ reaffirming the usefulness of electrophysiological STN identification for both awake and asleep DBS in Parkinson’s disease. However, the MER parameters used in this study were not able to distinguish the STN motor subdivision. Although the connectivity-derived mc-STN showed a significantly higher MUA compared with nmc-STN, the difference was not sufficient to reliably distinguish the two areas (ROC AUC 0.63). Previously, the motor-STN has been characterized by oscillatory and bursty spike patterns^21,22^ and a higher firing rate,^22^ while more irregular spike patterns^21^ and a higher burst rate^23^ were associated with STN areas outside of the motor-STN. Contrary to these findings, we did not find a difference in firing or burst rate between mc-STN and (slightly more anteriorly situated) nmc-STN, possibly due to the relatively small sample of single units in our data. This indicates that inside the dorsal STN, MUA and SUA alone are insufficient to determine the location relative to the area with the highest density of projections to cortical motor areas. Previous studies have found that the span of beta oscillations (as measured from MUA) within the surgical MER track correlates with motor outcome.^12^ The presence of beta oscillations (not evaluated in the current study) may therefore be used as an intraoperative surrogate for the STN motor area.^13^ However, it is also known that multiple factors may impede the level of MER resolution, as STN markers (MUA, SUA, beta, etc.) are affected by a variety of patient-specific and clinical variables (e.g. age, as we find here), in addition to the known effects of propofol anaesthesia and dopaminergic medication.^20^ Moreover, there is a considerable overlap of mc and nmc functional areas within the STN, and therefore, it may be possible to find electrophysiological activity indicating mc-STN even in areas that have predominant associative connectivity.^7^ For these reasons, there may be an upper limit to the reliability of MER to identify the mc-STN. Connectivity-derived segmentation could possibly provide a better insight into optimal electrode positioning compared with MER.

In contrast to 7 T MRI connectivity-derived segmentation, MER cannot be performed for preoperative targeting. Moreover, connectivity-derived segmentation is a non-invasive technique for visualizing the STN motor subdivision.^1,4-6,24^ Performing MER prolongs surgical time, especially when recordings need to be of sufficient duration to calculate beta power.^12,13^ Although many patients with Parkinson’s disease may benefit from MER-guided DBS, the percentage of good or excellent responders has not increased over the past 10 years. The threshold of 70% motor improvement has been reached only in a minority (30%) of patients.^14,25^ In the current study, all DBS electrodes were placed MER-guided, and 18% of them were located in the connectivity-derived motor-STN. These DBS electrodes achieved significantly better motor improvement (80%) compared with DBS electrodes placed in the remaining STN (52%).^7^ Therefore, connectivity-guided DBS electrode placement is likely to further improve DBS motor outcome in Parkinson’s disease.

### Limitations

Propofol is stopped during MER in our centre, limiting recording time. Recording lengths enabled the identification of few single units and were insufficient for beta power calculation, hampering comparison between groups using this electrophysiological measure to identify the motor-STN.^12,13^ Although 7 T MRI is not widely available, 3 T MRI may also be used for connectivity-derived STN segmentation,^1,26^ making the technique proposed here widely applicable.

## Conclusion

DBS in the connectivity-derived 7 T MRI STN motor segment produced a superior clinical outcome; however, MUA and SUA derived from MER did not accurately distinguish this subdivision within the STN. Further research is necessary to investigate how beta-oscillatory activity correlates with MRI connectivity and clinical outcome.

## Supplementary Material

fcad298_Supplementary_Data

## Data Availability

The data that support the findings of this study are available from the corresponding author upon reasonable request.
