# Supplementary material for: Dorsal subthalamic nucleus targeting in deep brain stimulation: microelectrode recording versus 7-Tesla connectivity
Source: Brain Commun. 2023 Nov 11;5(6):fcad298. doi: 10.1093/braincomms/fcad298 (PMC10664414; doi:10.1093/braincomms/fcad298)
Supplement: fcad298_Supplementary_Data [file fcad298_supplementary_data.pdf]

Supplementary Material

	Non-motor-connected STN	Motor-connected STN
Amplitude (mA)	2.2 ± 0.6	2.1 ± 0.7
Frequency (Hz)	129.5 ± 17.1	130 ± 0
Pulse width (microsec)	60 ± 0	60 ± 0
Side effect threshold	2.3 ± 0.8	2.7 ± 0.8
No. of electrodes w/o side effects until 3-3.5 mA	18/41 (44%)	2/9 (22%)

Supplementary table 1. Deep brain stimulation parameters compared between electrodes localized in the motor-connected STN versus the non-motor-connected STN. STN: subthalamic nucleus.
